# Burning the Candle at Both Ends: Have Exoribonucleases Driven Divergence of Regulatory RNA Mechanisms in Bacteria?

**DOI:** 10.1128/mBio.01041-21

**Published:** 2021-08-10

**Authors:** Daniel G. Mediati, David Lalaouna, Jai J. Tree

**Affiliations:** a School of Biotechnology and Biomolecular Sciences, University of New South Wales, Sydney, NSW, Australia; b Université de Strasbourg, CNRS, ARN UPR 9002, Strasbourg, France; National Institute of Child Health and Human Development (NICHD)

**Keywords:** 3′ UTR, Hfq, RNA decay, RNase J, evolution, small RNA

## Abstract

Regulatory RNAs have emerged as ubiquitous gene regulators in all bacterial species studied to date. The combination of sequence-specific RNA interactions and malleable RNA structure has allowed regulatory RNA to adopt different mechanisms of gene regulation in a diversity of genetic backgrounds. In the model *Gammaproteobacteria* Escherichia coli and Salmonella, the regulatory RNA chaperone Hfq appears to play a global role in gene regulation, directly controlling ∼20 to 25% of the entire transcriptome. While the model *Firmicutes* Bacillus subtilis and Staphylococcus aureus encode a Hfq homologue, its role has been significantly depreciated. These bacteria also have marked differences in RNA turnover. E. coli and Salmonella degrade RNA through internal endonucleolytic and 3′→5′ exonucleolytic cleavage that appears to allow transient accumulation of mRNA 3′ UTR cleavage fragments that contain stabilizing 3′ structures. In contrast, B. subtilis and S. aureus are able to exonucleolytically attack internally cleaved RNA from both the 5′ and 3′ ends, efficiently degrading mRNA 3′ UTR fragments. Here, we propose that the lack of 5′→3′ exoribonuclease activity in *Gammaproteobacteria* has allowed the accumulation of mRNA 3′ UTR ends as the “default” setting. This in turn may have provided a larger pool of unconstrained RNA sequences that has fueled the expansion of Hfq function and small RNA (sRNA) regulation in E. coli and Salmonella. Conversely, the exoribonuclease RNase J may be a significant barrier to the evolution of 3′ UTR sRNAs in B. subtilis and S. aureus that has limited the pool of RNA ligands available to Hfq and other sRNA chaperones, depreciating their function in these model *Firmicutes*.

## OPINION/HYPOTHESIS

Bacterial mRNAs have short half-lives, which allow rapid changes to the transcriptome in response to environmental cues. The processing of an mRNA may be directed, enhanced, or inhibited by regulatory small RNAs (sRNAs), which litter the transcriptome and have been extensively characterized in the model Gram-negative enteric bacteria Escherichia coli and Salmonella enterica serovar Typhimurium. Recently, it has become clear that regulatory sRNAs can be generated from a variety of sources in *Gammaproteobacteria*, including mRNA 5′ untranslated regions (UTRs) (1, 2), internal fragments of mRNA protein-coding sequences (CDSs) (1, 3), and an abundance of sRNAs transcribed or cleaved from the 3′ UTRs of mRNAs (references 4 and 5 and references below). Several RNA-binding proteins (RBPs) have been identified that play key roles in the biogenesis, function, and degradation of these sRNAs, and key among these are the chaperones Hfq and ProQ and endoribonuclease RNase E (*rne*) (6–9). These RBPs are well conserved among *Gammaproteobacteria* but are absent or have depreciated functions in the model Gram-positive *Firmicutes* Bacillus subtilis and Staphylococcus aureus. While sRNAs are abundant in the transcriptomes of these bacteria, there appear to be fundamental differences in the sources of sRNAs and mechanisms of regulation.

Here, we highlight some of the key differences in RNA metabolism between *Gammaproteobacteria* like E. coli and Salmonella and the model Gram-positive *Firmicutes* B. subtilis and S. aureus. Using examples, we propose that 3′ UTR sRNAs represent the latter stages of a stepwise continuum of increasing regulatory independence for mRNA 3′ UTRs. Finally, we propose that 5′→3′ exoribonuclease activity may be a barrier to this stepwise progression of regulatory 3′ UTR evolution in Gram-positive *Firmicutes*, which has contributed to the depreciation of sRNA chaperone Hfq and ProQ function.

## RNA METABOLISM HAS DIVERGED BETWEEN MODEL PROTEOBACTERIA AND FIRMICUTES

In both *Gammaproteobacteria* and the *Firmicutes* B. subtilis and S. aureus, bulk RNA turnover is carried out by a large multiprotein complex termed the RNA degradosome (10). The complex is often scaffolded by an endoribonuclease and associates with various accessory proteins that include exoribonucleases, helicases, and metabolic proteins that coordinate to efficiently degrade RNA. The decay of an mRNA transcript can occur through exoribonucleases or through internal cleavage by endoribonucleases that make the transcript vulnerable to either 5′→3′ or 3′→5′ exoribonucleolytic degradation. The composition of the degradosome is also highly variable between *Gammaproteobacteria* and *Firmicutes*. While the major scaffold for the RNA degradosome in *Gammaproteobacteria* is the endoribonuclease RNase E, this RBP is not present in S. aureus and B. subtilis, which instead possess the endoribonuclease RNase Y, which appears to act as a scaffold for the RNA degradosome complex. RNase E is not completely absent from all Gram-positive bacteria. Those that contain a high-GC% genome, such as *Streptomyces* species, possess a homolog of RNase E (*rns*) that contains the conserved catalytic *N*-terminal region of the protein and can functionally substitute for *rne* in E. coli (11). However, the canonical mechanism of RNA degradation within the majority of Gram-positive bacteria appears to be endonucleolytic cleavage by either RNase Y or RNase III, subsequently allowing 3′→5′ exoribonucleases such as PNPase and RNase R (12) and 5′→3′ exoribonucleases such as RNase J1 and J2 to process the RNA substrates. This is in stark contrast to the Gram-negative enteric bacteria like E. coli and Salmonella, which predominantly rely on 3′→5′ exoribonucleases to degrade fragmented RNA processed by internal endoribonucleolytic cleavage events. *Gammaproteobacteria* largely lack the 5′→3′ exoribonucleolytic activity (RNase J) that is commonly associated with the RNA degradosome complex of Gram-positive *Firmicutes*. There has recently been evidence of a 5′→3′ exoribonuclease within E. coli (13); however, RNase AM has only been identified to process the last few nucleotides at the 5′ ends of the 5S, 16S, and 23S rRNA (14).

The 5′→3′ exoribonucleolytic activity is well described in eukaryotes but was thought to not exist in prokaryotes, until the discovery of RNase J1/J2 in B. subtilis (15). Both exoRNases are bifunctional and also possess endonuclease activity (16, 17). Characterization reports show that RNase J1 is the more active and principal exoribonuclease of the pair, as an RNase J1 depletion increased the abundance of ∼20% of mRNA transcripts (18). Additionally, in a B. subtilis RNase J1 deletion strain (Δ*rnjA*), the 3′-terminal RNA fragments (containing the transcription terminator) of more than 50% of mRNAs were increased, highlighting the importance of RNase J1 in degradation of 3′-terminal RNA decay intermediates (19).

Chaperones play critical roles in facilitating sRNA regulation by increasing the association rate of sRNA-mRNA interactions to physiologically useful rates. The sRNA chaperone Hfq binds many sRNAs through their poly(U) tail and acts as a matchmaker within Gram-negative bacteria to anneal sRNAs with complementary mRNAs, while ProQ binds to the 3′ UTR of mRNAs to protect the free mRNA 3′ end against 3′→5′ exoribonucleolytic degradation. Some Gram-positive organisms, such as Streptococcus pneumoniae, do not possess an RNA-binding chaperone homologous to either Hfq or ProQ (20). In fact, ProQ appears to generally be absent in *Firmicutes* (21, 22). In Listeria monocytogenes, it appears that Hfq contributes to pathogenesis and has a role in certain stresses such as osmotic and amino acid-limiting conditions (23); however, an *hfq* deletion showed no major sRNA expression changes (24). In S. aureus, the expression and role of Hfq appear to be strain specific, and the deletion does not seem to have the highly pleiotropic effects seen in E. coli *hfq* mutants (25, 26). The exception to the rule may be Clostridioides difficile where deletion of *hfq* affects expression of 224 genes (5% of genes, compared with 785 [18%] of genes in Salmonella Typhimurium [27]) and has pleiotropic effects on sporulation, growth, morphology, and stress responses (28). Hfq binds and stabilizes a subset of sRNAs in C. difficile (28, 29), and, importantly for the discussion below, recent Hfq RNA immunoprecipitation sequencing (RIP-seq) experiments have identified 18 3′ UTR-encoded sRNAs, including five type II 3′ UTR sRNAs (29). The C. difficile transcriptome may encode between 42 and 251 regulatory sRNAs (29, 30), and the relative proportion of the total sRNA repertoire that is generated from 3′ UTRs is unclear, but it seems that the role of Hfq has been expanded compared to other *Firmicutes*. Like B. subtilis and S. aureus, C. difficile encodes RNase J1 (49.8% and 52.1% amino acid identity to S. aureus and B. subtilis, respectively) and is expected to have 5′→3′ exoribonuclease activity.

In the section below, we propose that there exists a continuum of regulatory 3′ UTR independence and that the 5′→3′ exoribonuclease activity of RNase J in B. subtilis and S. aureus may be a barrier to evolution of independent regulatory 3′ UTR sRNAs along this continuum.

## A CONTINUUM OF REGULATORY sRNA EVOLUTION

Multiple pathways likely exist for the evolution of regulatory sRNAs within the transcriptome (31–38). However, for the evolution of any regulatory RNA species, the first steps are transcription and stabilization. Without both, there would be limited opportunity for interactions with target RNAs and for gaining a foothold on the ladder to positive selection. Small RNAs in E. coli largely appear in ancestral genomes before their cognate target mRNA binding sites, suggesting that sRNAs are first produced and then drive evolution of target mRNAs (39). Regulatory RNA species are suggested to have low expression levels that increase as the sRNA becomes integrated into the host regulatory network (35). Pervasive transcription has been suggested as a source of regulatory RNAs, and this occurs in most bacterial genomes but is limited by H-NS, RNase III, and Rho terminator (40). RNA surveillance within the cell also prevents accumulation of aberrant transcripts that lack stabilizing features like a structured 3′ or 5′ end. The most abundant stable RNA species within the cell that are not subject to the evolutionary constraints exerted by CDS or RNA structure (e.g., rRNA, tRNA, and transfer-messenger RNA [tmRNA]) are the UTRs of mRNAs. The 3′ UTRs of mRNAs have been proposed to be a “playground” for sRNA evolution and may serve as a major reservoir of unconstrained RNA sequence for the evolution of regulatory RNA (41, 42).

## mRNA 3′ UTRs THAT ACT IN *CIS*

*cis-*acting regulatory 3′ UTRs are well-documented in eukaryotes and modulate the expression of the upstream CDS. A limited number of regulatory mRNA 3′ UTRs have also been identified in bacteria (i.e., that are not independent or processed transcripts). The simplest arrangement of a regulatory UTR and target mRNA is found in S. aureus where the 3′ UTR of *icaR* mRNA loops on itself (or between *icaR* mRNAs) and base pairs to the ribosome-binding site (RBS) of its own 5′ UTR to block translation and promote RNase III-dependent degradation (43). In this arrangement, the mRNA UTRs act in *cis*, and a single transcript serves as both regulatory RNA and target RNA. A similar regulatory interaction, with the opposite regulatory effect, has been described in B. subtilis, where an interaction between the 5′ UTR and 3′ UTR of *hbs* mRNA occludes an RNase Y cleavage site in the 5′ UTR and stabilizes the mRNA (44). The relative simplicity of this regulation suggests that more examples of *cis*-acting regulatory mRNA 3′ UTRs may exist and control gene regulation in Gram-positive *Firmicutes*. This is supported by the observation that 3′ UTRs are more variable than CDSs when species within the same genus are compared, and variation in the 3′ UTR appears to be partly responsible for the differences in expression levels of orthologous coding sequences (45).

## mRNA 3′ UTRs THAT ACT IN *TRANS*

A slightly more complex variation where mRNA 3′ UTRs act in *trans* is found in the Gram-positive bacterium Listeria monocytogenes. The mRNA 3′ UTR of listeriolysin O encoded by *hly* base pairs with the 5′ UTR of the listeriolysin O chaperone mRNA *prsA2* (46). This mRNA-mRNA base-pairing between the *hly* 3′ UTR and *prsA2* 5′ UTR blocks RNase J1 exonucleolytic attack of the *prsA2* 5′ end, stabilizing the chaperone transcript and listeriolysin O protein (46). The *hly* 3′ UTR is an elegant example of a dual-function mRNA that provides coherent regulatory connections between functionally related mRNA UTRs. Many mRNA 3′ UTRs in Gram-positive bacteria are long, and in S. aureus, more than 30% of mRNA 3′ UTRs are greater than 100 nucleotides (nt) (43) (compared to 15% in E. coli [47]), suggesting that *trans*-acting regulatory 3′ UTRs could be a widespread mechanism of regulation in *Firmicutes*. An advantage is that *trans*-acting regulatory 3′ UTRs like the *hly* 3′ UTR are protected from the 5′→3′ exoribonucleolytic processing of RNase J (to the extent that the mRNA is protected).

## PROCESSED 3′ UTRs THAT ACT IN *TRANS* (TYPE II 3′ UTR sRNAs)

Many examples have now been described where the dual regulatory and coding functions of mRNAs are separated into distinct RNA species through processing or independent transcription of regulatory 3′ UTRs. In S. aureus, the regulatory 3′ UTR-derived sRNA RsaC is generated by endoribonucleolytic cleavage of the polycistronic *mntABC-rsaC* transcript by RNase III (48). RsaC is, in effect, the long 3′ UTR of the *mntABC* mRNA (encoding a manganese transporter) and while they share a transcriptional activation signal (repressed in the presence of Mn^2+^ by MntR), RNase III cleavage separates these transcripts so that they have independent fates within the cell. RsaC retains mRNA targets that are functionally coherent with the *mntABC-rsaC* operon, repressing the Mn^2+^-dependent superoxide dismutase (SodA), and other metal-dependent pathways. RNase III cleavage of *mntABC-rsaC* generates a free 5′ end that should render RsaC highly susceptible to RNase J exonucleolytic attack. However, a notable feature of RsaC is the 25-nt stem-loop that sequesters the 5′ end in a duplex preventing exoribonuclease attack and stabilizes the 3′ UTR-derived sRNA (48).

A further Gram-positive 3′ UTR-derived sRNA has been described in Streptomyces coelicolor. The 3′ UTR of *sodF* mRNA, encoding an Fe-containing superoxide dismutase, is processed to release the 90-nt s-SodF sRNA. s-SodF destabilizes the mRNA for Ni-containing superoxide dismutase (*sodN*) allowing coordination between these functionally related genes (49). Like S. aureus, S. coelicolor encodes RNase J, and to prevent rapid degradation, the 5′ end of s-SodF also folds into a 20-nt stem-loop, sequestering the 5′ from exonuclease attack (49). Protective 5′ structures would be expected in 3′ UTR-derived sRNAs of any bacterium with robust 5′→3′ exoribonucleolytic activity, which may form an evolutionary barrier for widespread evolution of the regulatory 3′ UTR sRNAs in RNase J-encoding bacteria.

In contrast to Gram-positive bacteria, for which relatively few 3′ UTR-derived sRNAs have been described so far, Gram-negative organisms appear to be replete with these regulatory sRNA species that are released from mRNAs by endonucleolytic cleavage (5, 50–52). In the Gram-negative pathogen Vibrio cholerae, the 3′ UTR sRNA OppZ is encoded at the 3′ end of the *oppABCDF* operon (encoding an oligopeptide transporter). RNase E cleavage after the *oppF* stop codon releases OppZ from the parent transcript. In a regulatory circuit that parallels the 5′ UTR-3′ UTR looping autoregulation of *icaR* in S. aureus, OppZ binds the upstream RBS of *oppB* to silence expression and control the cellular levels of the precursor *oppBCDF* transcript through a feedback loop (53). The OppZ regulon is narrow and appears to regulate only the *oppBCDF* operon. Other 3′ UTR-derived sRNAs control expression of genes that are functionally linked to the protein encoded within the mRNA, indicating that 3′ UTR-derived sRNAs can act to coordinate protein expression between functionally related mRNAs. A recent example is the 3′ UTR sRNA *narS*, derived from the *narK* mRNA encoding a nitrate (NO_3_^−^) transporter in Salmonella. The *narK* mRNA is expressed during anaerobic respiration (54, 55), and RNase E cleavage releases NarS from the mRNA (56). NarS negatively regulates the nitrite (NO_2_^−^) transporter *nirC*, which is located within the *nirBDC-cysG* operon and controls cytoplasmic nitrate levels during anaerobic growth (54). NarS is able to repress *nirC* through a perfect 14-nt interaction that blocks the RBS of *nirC*. Similar to OppZ, NarS appears to regulate a single target, *nirC*, allowing suboperonic coordination between *narK* and *nirC* mRNAs.

## INDEPENDENTLY TRANSCRIBED 3′ UTRs THAT ACT IN *TRANS* (TYPE I 3′ UTR sRNAs)

Transcription from an internal promoter incorporates further regulatory independence and allows integration of new transcriptional regulatory signals. This should correlate with increasingly diverse regulons as the regulatory RNA becomes more integrated into the broader regulatory network (57). Notably, many 3′ UTR sRNAs that are transcribed from an independent promoter are still processed by RNases to release the mature sRNA, and many require the processed 5′ monophosphate for activity, presumably for activation of the RNase E sensor pocket (58). One example of independent transcription of an mRNA 3′ UTR is DapZ in Salmonella. The independent gene-internal promoter of DapZ is located upstream of the stop codon of the essential lysine biosynthetic *dapB* gene. Transcription from the DapZ promoter is controlled by the transcriptional activator HilD (59). Overexpression of DapZ in Salmonella showed ∼15 differentially expressed mRNAs (such as the *glt* operon and *serA* and *cycA* mRNAs), and further experiments confirmed that DapZ negatively regulates the synthesis of major ABC transporters through the repression of both *dpp* and *opp* mRNAs (59). Like type II sRNAs, DapZ also shows evidence of internal cleavage; however, these cuts appear to be nonproductive, as they remove the R1 seed that allows it to commandeer the GcvB regulon (59).

In the Gram-positive bacterium Lactococcus lactis, the 3′ UTR of the arginine responsive transcriptional factor *argR* encodes a 66-nt 3′ UTR sRNA termed ArgX, which is transcribed from an ArgR-responsive promoter within its own 3′ UTR. Both ArgX and ArgR function in a negative feed-forward loop to repress the *arc* arginine catabolism operon during arginine limitation (60). ArgX does not have a structured 5′ end to prevent RNase J exonucleolytic attack. In B. subtilis, RNase J activity is inhibited by the 5′ triphosphate present on primary transcripts (61), and this may protect the unstructured ArgX 5′ from degradation. These results suggest an alternative pathway for stabilization of 3′ UTRs in bacteria with 5′→3′ exonuclease activity: acquisition of an internal promoter and a 5′ triphosphate “cap.”

## DIVERGENCE OF RNA SURVEILLANCE MACHINERY MAY EXPLAIN DIVERGENCE OF REGULATORY RNA MECHANISMS

**FIG 1:**
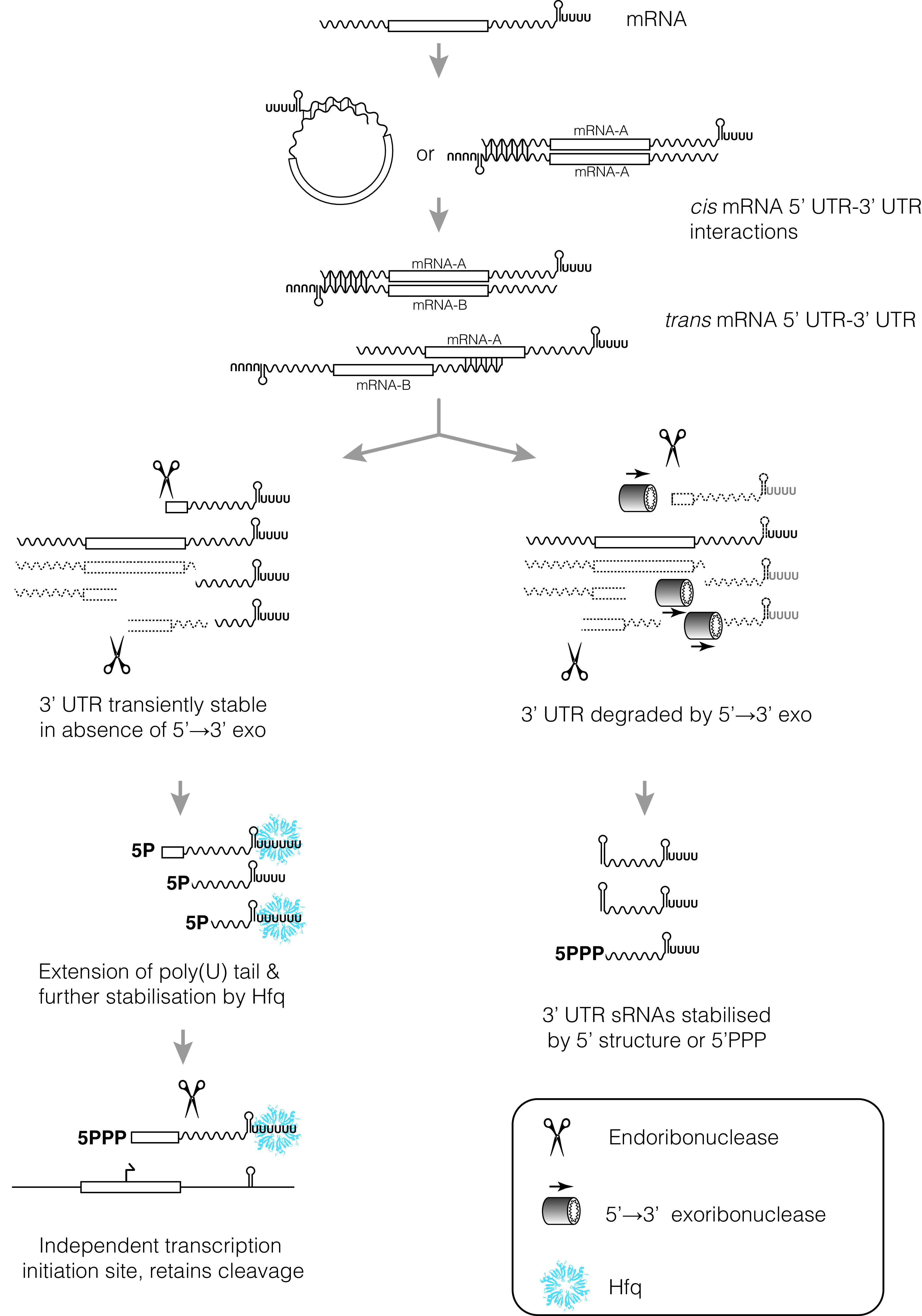
A continuum of regulatory 3′ UTR independence (see the text for a detailed description and examples). The UTRs of bacterial mRNAs are highly variable and contribute to expression of the coding sequence. Selected 3′ UTRs have been found to interact with *cis*-encoded 5′ UTRs, *trans*-encoded 5′ UTRs, and CDSs to control mRNA stability. The regulatory functions of many 3′ UTRs appear to have been physically separated from the mRNA. For bacteria that lack 5′→3′ exoribonuclease activity, cleaved 3′ UTRs will be stabilized by the intrinsic terminator alone (left branch). This may allow cleaved 3′ fragments to acquire additional stabilizing features, like an extended poly(U) tail that recruits the match-making sRNA chaperone, Hfq. As the regulatory 3′ UTR acquires more mRNA targets, additional regulatory inputs (like internal transcription start sites) may allow further separation of 3′ UTR and mRNA functions. (Right branch) In bacteria that encode 5′→3′ exoribonuclease activity, mRNA 3′ UTRs are efficiently degraded from the 5′ end. These 3′ UTRs must first acquire stabilizing 5′ structures (stems that sequester the 5′ end), or internal promoters that deposit protective 5′ triphosphates, before they are stabilized.

Collectively, the progression from *cis* interactions between mRNA 5′ and 3′ UTRs, to *trans* interactions between mRNA UTRs, to cleavage of *trans*-acting regulatory 3′ UTRs, and to independent gene-internal transcription of *trans*-acting 3′ UTRs may represent a continuum of sRNA evolution (Fig. 1). While there are clearly other sources of stable RNA for the evolution of regulatory sRNAs (e.g., pervasive transcription, genome rearrangements, tRNA spacers, and anti-termination regulated promoters), we propose that the stepwise acquisition of 3′ UTR sRNAs outlined here may serve as a major pathway for the evolution of regulatory sRNA, particularly in *Gammaproteobacteria*. If mRNA 3′ UTRs are a substantial evolutionary source of sRNAs, it may explain why sRNAs and chaperones appear so different between Gram-positive and Gram-negative bacteria.

**FIG 2:**
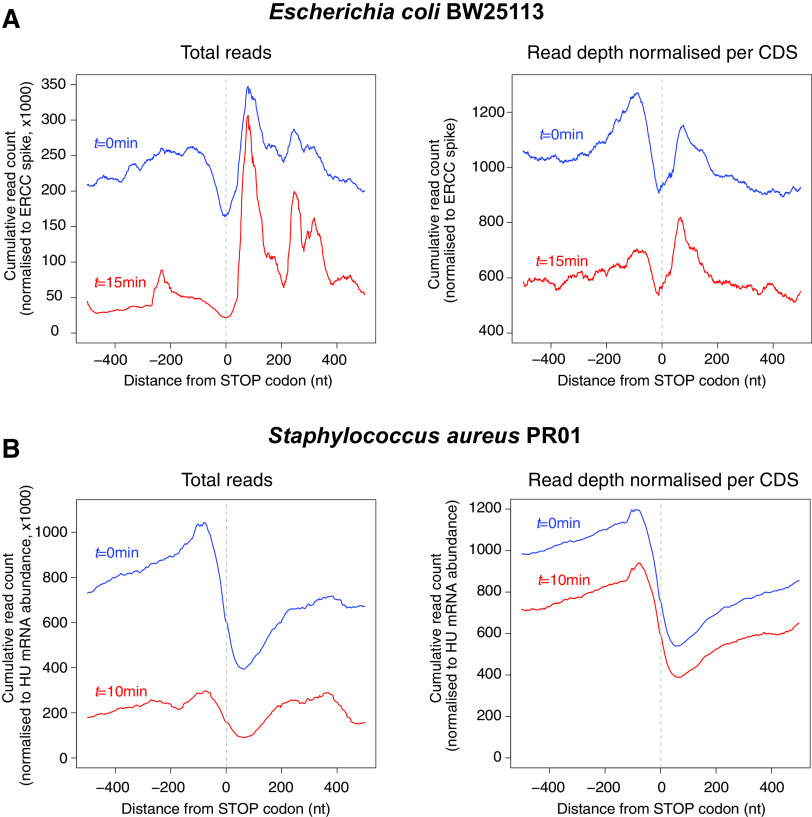
mRNA 3′ UTRs in E. coli are more stable than the upstream coding sequence and more abundant than S. aureus 3′ UTRs. (A) Transcriptome-wide RNA stability data for E. coli stop codons at 0 and 15 min after rifampin treatment are compared (data processed from reference 66 [ENA accession no. PRJEB21982]). (Left) Cumulative read count of RNA-seq reads (normalized to ERCC spike) mapping within 500 nt of mRNA stop codons at 0 min (blue) and 15 min (red) after rifampin treatment. (Right) To account for highly stable regulatory 3′ UTR sRNAs that may disproportionately contribute to the strong 3′ UTR peak in total read counts (left), the data for each stop codon were normalized to the local maxima (each contributing to a maximum value of 1). (B) As for panel A, except that transcriptome-wide RNA stability data for S. aureus stop codons were compared for 0 and 10 min after rifampin treatment (data processed from reference 67 [NCBI GEO accession no. GSE68811]). Stability data are normalized to the abundance of HU mRNA as per reference 67.

**FIG 3:**
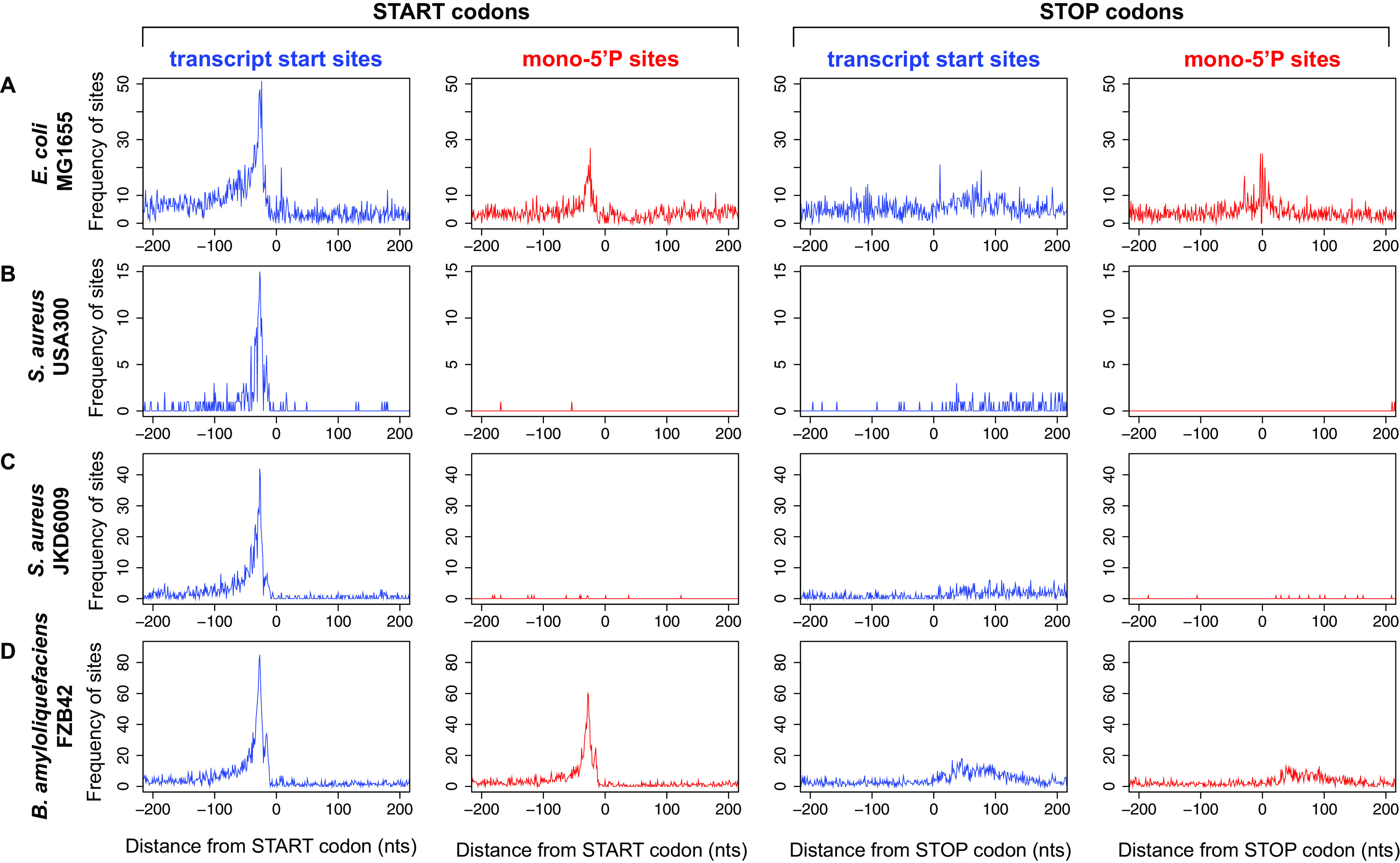
RNA 5′ ends identified by differential RNA-seq in E. coli and S. aureus. Differential RNA-seq captures transcription start sites (triphosphorylated RNA 5′ ends; blue plots) and processing sites (monophosphorylated RNA 5′ ends; red plots). For each strain indicated on the left, the cumulative frequency of transcription start sites (red) or processing sites (blue) is plotted relative to the start codon or the stop codon (indicated above the graphs) for all CDSs within the genome. Each transcription start site or processing site contributes equally to the frequency plot (i.e., each site = 1). (A) E. coli MG1655 dRNA-seq data processed from reference 68 (NCBI GEO accession no. GSE55199). (B) S. aureus USA300 dRNA-seq data processed from reference 69 (ENA accession no. PRJEB23980). (C) S. aureus JKD6009 dRNA-seq data (NCBI GEO accession no. GSE158830). (D) Bacillus amyloliquefaciens FZB42 dRNA-seq data (NCBI GEO accession no. GSE66681). For JKD6009 data, reads were aligned using Novoalign and read counts were mapped using pyCRAC software (70). For all data sets, TSS and processing sites were identified using the tss_ps module of ANNOgesic (71) on default settings.

Many Gram-negative organisms lack a 5′→3′ exoribonuclease and rely on endoribonucleolytic cleavage (such as RNase E) and 3′→5′ exoribonucleases to degrade RNA within the cell. In the model Gram-negative bacteria E. coli and Salmonella Typhimurium, RNase E is the major endoribonuclease and cleaves RNA on average every 175 nt (56), generating short RNA fragments that are degraded by exonucleolytic processing from the free 3′ end by PNPase, RNase R, and RNase II. Protective RNA structures at the 3′ end of RNA transcripts inhibit exonucleolytic attack and can lead to differential stability of genes within polycistronic transcripts or internal RNA fragments (47). It seems likely that the last RNase E cleavage fragment of most mRNAs would also have increased stability. This fragment is protected by an intrinsic terminator (or another 3′ structure for Rho-terminated transcripts [47]), and these 3′ fragments might be expected to have a slightly longer half-life in bacteria lacking 5′→3′ exonuclease activity. Analysis of RNA stability 200 nt before and after stop codons indicates that 3′ UTRs in E. coli are more stable than the upstream coding sequence (Fig. 2A). In contrast, the 3′ UTRs of S. aureus are less abundant than the upstream coding sequence even at steady state (Fig. 2B). This is supported by differential transcriptome sequencing (dRNA-seq) data that identify transcription start sites (TSS; triphosphorylated 5′ ends) and processing sites (PS; monophosphorylated 5′ ends). In E. coli, processing sites are abundantly detected at stop codons (Fig. 3A), likely reflecting the increased stability of 3′ UTRs or 3′ terminal mRNA cleavage fragments. Similar RNase E-dependent cleavage sites at stop codons has been described in *S.* Typhimurium (56). In S. aureus and Bacillus amyloliquefaciens, processing sites are less abundant at stop codons (relative to primary transcription at start sites), suggesting that independently transcribed or processed 3′ UTRs are less abundant in these Gram-positive organisms (Fig. 3B to D).

In E. coli and Salmonella, it is plausible that this pool of 3′ mRNA fragments provides a source of transiently stable, unconstrained RNA sequence space for the selection and evolution of regulatory RNA features. It follows that RNase J exoribonucleolytic activity in many Gram-positive organisms may pose a significant barrier to evolution of new 3′ UTR regulatory RNAs. Without first acquiring stabilizing 5′ structures, these 3′ RNA fragments are rapidly degraded and would have limited opportunity to gain a foothold on the ladder to positive selection.

The functional importance of Hfq appears to be significantly expanded in many Gram-negative organisms, and we propose that this may be linked to the availability of “stable” 3′ UTR degradation intermediates (Fig. 1). Hfq binds many sRNAs to a proximal RNA binding surface that recognizes the poly(U) tail of the intrinsic terminator. Hfq binding appears to be associated with transcripts that carry a slightly longer poly(U) tract, which provides some selectivity within a pool of hundreds of transcripts that utilize intrinsic termination (62–64). Extension of the poly(U) tail of 3′ UTR fragments, and association with Hfq, may provide a secondary step on the ladder to functional sRNA. Hfq binding would further stabilize the 3′ end of the 3′ UTR fragment by occluding 3′→5′ exonucleases and allow selection of mRNA seed complementarity. This may be one of the reasons that Hfq function has been depreciated in many Gram-positive organisms that encode 5′→3′ exonucleases: as the final endonucleolytic cleavage fragment would not have increased stability by default, there may not exist a ready pool of 3′ UTR fragments to positively select through stabilization and target annealing. In microorganisms that efficiently degrade 3′ UTR fragments, Hfq may be deprived of an important source of RNA to select for advantageous sRNA-mRNA interactions, leading to the depreciation of Hfq function.

An analogous scenario has occurred in many Gram-positive *Firmicutes* where the function of Rho terminator may have been depreciated because the pioneering ribosome lags behind the elongating RNA polymerase, exposing the nascent transcript to premature Rho interactions and potentially toxic transcription termination (65). In contrast, in Gram-negative *Proteobacteria*, the pioneering ribosome remains closely associated with the elongating RNA polymerase and prevents pervasive Rho association and premature termination. For both Rho and Hfq, the availability of RNA targets in many Gram-positive organisms may have selected against their widespread incorporation into gene regulatory circuits (albeit an overabundance of targets for Rho and paucity of targets for Hfq).

## CONCLUSIONS

All bacteria appear to use regulatory RNA to control gene expression posttranscriptionally; however, it is clear that differences exist in the distribution and importance of RNA chaperones that facilitate sRNA-mRNA interactions. We propose that mRNA 3′ UTRs are a major evolutionary source for regulatory sRNAs and highlight some of the potential intermediate stages of 3′ UTR sRNA evolution. In addition, we propose that the lack of 5′→3′ exoribonucleases in E. coli and Salmonella allows transient stabilization of cleaved mRNA 3′ UTRs, which has provided abundant raw materials for the evolution of 3′ UTR sRNAs and the expansion of sRNA regulatory networks. This in turn has centralized the function of sRNA chaperones like Hfq in E. coli and Salmonella as the sRNA network has expanded. In Gram-positive *Firmicutes* that encode a 5′→3′ exoribonuclease (RNase J), cleaved 3′ UTRs are rapidly degraded. In these bacteria, 3′ UTR sRNAs must first acquire 5′ stems or internal transcription start sites before they are stabilized. We propose that RNase J represents a major evolutionary barrier to the expansion of the 3′ UTR sRNA network and has depreciated the function of the sRNA chaperones Hfq and ProQ.

Some testable predictions arise from the above. (i) As the relative importance of Hfq for global gene regulation is uncovered in more bacteria, the size of the Hfq regulon should be negatively correlated with the presence of a functional RNase J. (ii) Endoribonuclease cleavage of 3′ UTR sRNAs should be less prevalent in RNase J-encoding bacteria. These bacteria may make more widespread use of regulatory mRNA 3′ UTRs (like *hly* mRNA in *Listeria*) that are not cleaved to protect the 5′ end of the UTR. Evolution of internal transcription start sites should be the preferred mechanism of 3′ UTR release, as this provides a protective 5′ triphosphate.
